# Crystal structure of 2-acetyl-5-(3-methoxyphenyl)-3,7-dimethyl-5*H*-1,3-thiazolo[3,2-*a*]pyrimidine-6-carboxylate

**DOI:** 10.1107/S1600536814023162

**Published:** 2014-10-29

**Authors:** N. L. Prasad, M. S. Krishnamurthy, H. Nagarajaiah, Noor Shahina Begum

**Affiliations:** a Department of Studies in Chemistry, Bangalore University, Bangalore 560 001, Karnataka, India

**Keywords:** crystal structure, thia­zolo­pyrimidine derivative, C—H⋯S inter­actions, π–π inter­actions

## Abstract

In the title mol­ecule, C_20_H_22_N_2_O_4_S, the pyrimidine ring is in a flattened half-chair conformation and the 3-meth­oxyphenyl substituent is in an axial arrangement. The thia­zole ring forms a dihedral angle of 81.3 (1)° with the benzene ring. In the crystal, weak C—H⋯S inter­actions link mol­ecules into chains along [001]. In addition, there are π–π inter­actions between inversion-related thia­zole rings with a centroid–centroid distance of 3.529 (2) Å. The ethyl group was refined as disordered over two sets of sites with an occupancy ratio of 0.52 (3):0.48 (2).

## Related literature   

For pharmacological and biological properties of pyrimidine derivatives, see: Alam *et al.* (2010*a* ▶,*b* ▶). For the therapeutic potential of thia­zolo­pyrimidine derivatives, see: Zhi *et al.* (2008 ▶). For related crystal structures, see: Jotani *et al.* (2010 ▶); Nagarajaiah *et al.* (2012 ▶).
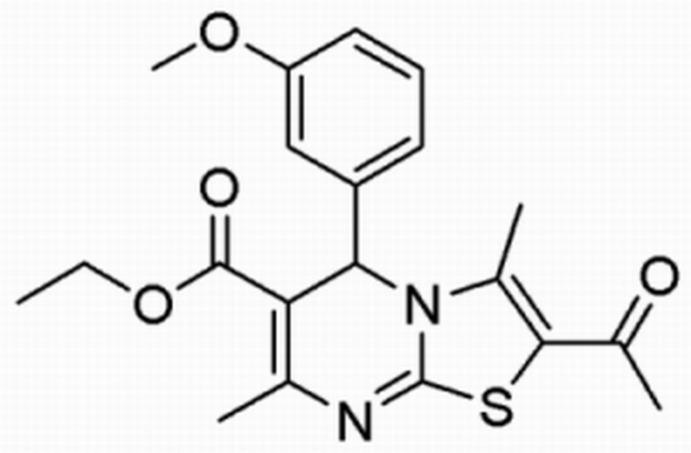


## Experimental   

### Crystal data  

C_20_H_22_N_2_O_4_S
*M* *_r_* = 386.46
Triclinic, 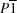

*a* = 8.281 (3) Å
*b* = 9.680 (4) Å
*c* = 12.821 (5) Å
α = 76.423 (10)°
β = 86.308 (10)°
γ = 74.641 (11)°
*V* = 963.3 (7) Å^3^
*Z* = 2
Mo *K*α radiation
μ = 0.20 mm^−1^
*T* = 100 K
0.18 × 0.16 × 0.16 mm

### Data collection  

Bruker SMART APEX CCD-detector diffractometer
Absorption correction: multi-scan (*SADABS*; Bruker, 1998 ▶) *T*_min_ = 0.966, *T*_max_ = 0.969
7744 measured reflections
4170 independent reflections
2529 reflections with *I* > 2σ(*I*)
*R*_int_ = 0.040

### Refinement  

*R*[*F* ^2^ > 2σ(*F* ^2^)] = 0.066
*wR*(*F* ^2^) = 0.173
*S* = 0.93
4170 reflections
263 parameters
H-atom parameters constrained
Δρ_max_ = 0.34 e Å^−3^
Δρ_min_ = −0.39 e Å^−3^

### 

Data collection: *SMART* (Bruker, 1998 ▶); cell refinement: *SAINT* (Bruker, 1998 ▶); data reduction: *SAINT*; program(s) used to solve structure: *SHELXS97* (Sheldrick, 2008 ▶); program(s) used to refine structure: *SHELXL97* (Sheldrick, 2008 ▶); molecular graphics: *ORTEP-3 for Windows* (Farrugia, 2012 ▶) and *PLATON* (Spek, 2009 ▶); software used to prepare material for publication: *WinGX* (Farrugia, 2012 ▶).

## Supplementary Material

Crystal structure: contains datablock(s) global, I. DOI: 10.1107/S1600536814023162/lh5730sup1.cif

Structure factors: contains datablock(s) I. DOI: 10.1107/S1600536814023162/lh5730Isup2.hkl

Supporting information file. DOI: 10.1107/S1600536814023162/lh5730Isup3.cml

**Fig. 1.**
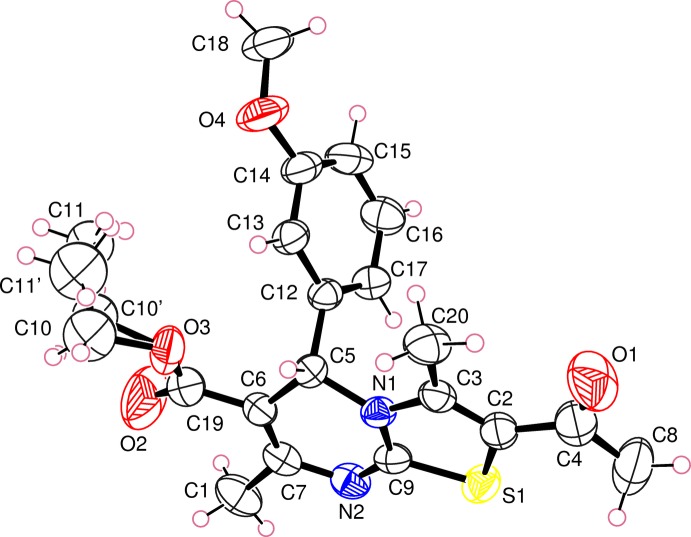
The molecular structure of the title compound with displacement ellipsoids drawn at the 50% probability level. H atoms are presented as small spheres of arbitrary radius. The primed atoms indicate the disorder.

. DOI: 10.1107/S1600536814023162/lh5730fig1.tif
The mol­ecular structure of the title compound with displacement ellipsoids drawn at the 50% probability level. H atoms are presented as small spheres of arbitrary radius. The primed atoms indicate the disorder.

**Fig. 2.**
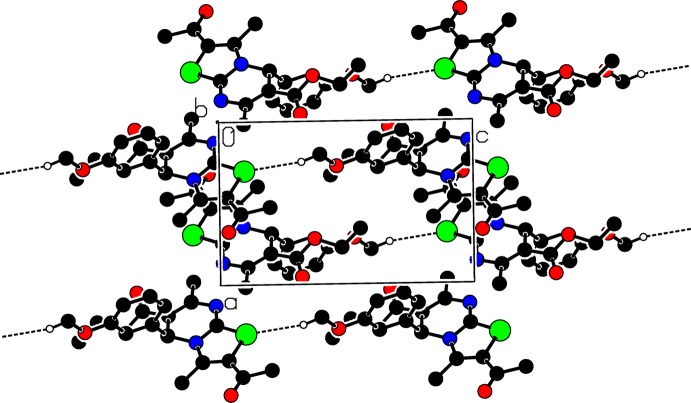
Part of the crystal structure with weak C—H···S interactions shown as dashed lines. H atoms not involved in hydrogen bonding have been excluded.

. DOI: 10.1107/S1600536814023162/lh5730fig2.tif
Part of the crystal structure with weak C—H⋯S inter­actions shown as dashed lines. H atoms not involved in hydrogen bonding have been excluded.

CCDC reference: 1030239

Additional supporting information:  crystallographic information; 3D view; checkCIF report

## Figures and Tables

**Table 1:** Hydrogen-bond geometry (, )

*D*H*A*	*D*H	H*A*	*D* *A*	*D*H*A*
C18H18*B*S1^i^	0.98	2.87	3.822(3)	162

Symmetry code: (i) 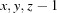.

## supplementary crystallographic information

### S1. Comment

Pyrimidine derivatives are of interest because of their pharmacological properties (Alam *et al.*, 2010*a*). A thiazole ring fused to a pyrimidine ring resulting in thiazolopyrimidine is found to possess remarkable biological activities such as antiviral, anticancer, anti-inflammatory and anti-hypertensive properties (Alam *et al.*, 2010*b*). In addition, thiazolopyrimidine derivatives possess therapeutic potential (Zhi *et al.*, 2008). Herein, we report the crystal structure of the title compound. The molecular structure of the title compound is shown in Fig. 1. The mean-plane of the 3-methoxy phenyl group adopts a *syn* periplanar conformation with respect to the C5—H5 bond of the pyrimidine ring. The pyrimidine ring is in a flattened half chair conformation with atoms N1 and C5 displaced by -0.082 (3) and 0.189 (4)Å, respectively from the mean plane of the other four atoms (N2/C6/C7/C9). The 3-methoxy phenyl substituent bonded to atom C5 is in an axial position. The ethyl group was refined as disordered over two sets of sites with an occupancy ratio of 0.52 (3):0.48 (2). The bond lengths and angles in the title compound are in good agreement with the corresponding bond distances and angles reported in closely related structures (Nagarajaiah *et al.*, 2012; Jotani *et al.*, 2010). In the crystal, weak C—H···S interactions link molecules into chains along [001] (Fig. 2). In addition, there are π···π interactions between inversion related thiazole rings with a centroid–centroid distance of 3.529 (2) Å.

### S2. Experimental

A mixture of 4-(3-methoxy-phenyl)-6-methyl-2-thioxo-1,2,3,4-tetrahydro-pyrimidine-5-carboxylic acid ethyl ester (10 mmol) and 3-chloro-2,4-pentanedione (10 mmol) was refluxed in dry ethanol (20 mmol) for 12 h. The excess of solvent was distilled off and the solid hydrochloride salt that separated was collected by filtration, suspended in water and neutralized by aqueous sodium carbonate solution to yield the free base. The solution was filtered, the solid washed with water, dried and recrystallized from ethyl acetate to give the title compound (76% yield, mp 380 K). The compound was recrystallized by slow evaporation from 1:1 mixture of ethyl acetate and methanol , yielding pale-yellow single crystals suitable for X-ray diffraction studies.

### S3. Refinement

The H atoms were placed at calculated positions in the riding-model approximation with C—H = 0.95° A, 1.00 Å and 0.96 Å for aromatic, methyne and methyl H-atoms respectively, with *U*_iso_(H) = 1.5*U*_eq_(C) for methyl H atoms and *U*_iso_(H) = 1.2*U*_eq_(C) for other hydrogen atoms.

### Crystal data

**Table:**

C_20_H_22_N_2_O_4_S	*Z* = 2
*M**_r_* = 386.46	*F*(000) = 408
Triclinic, *P*1	*D*_x_ = 1.332 Mg m^−^^3^
Hall symbol: -P 1	Mo *K*α radiation, λ = 0.71073 Å
*a* = 8.281 (3) Å	Cell parameters from 2529 reflections
*b* = 9.680 (4) Å	θ = 2.2–27.0°
*c* = 12.821 (5) Å	µ = 0.20 mm^−^^1^
α = 76.423 (10)°	*T* = 100 K
β = 86.308 (10)°	Block, yellow
γ = 74.641 (11)°	0.18 × 0.16 × 0.16 mm
*V* = 963.3 (7) Å^3^	

### Data collection

**Table:**

Bruker SMART APEX CCD-detector diffractometer	4170 independent reflections
Radiation source: fine-focus sealed tube	2529 reflections with *I* > 2σ(*I*)
Graphite monochromator	*R*_int_ = 0.040
ω scans	θ_max_ = 27.0°, θ_min_ = 2.2°
Absorption correction: multi-scan (*SADABS*; Bruker, 1998)	*h* = −10→10
*T*_min_ = 0.966, *T*_max_ = 0.969	*k* = −12→12
7744 measured reflections	*l* = −11→16

### Refinement

**Table:**

Refinement on *F*^2^	Primary atom site location: structure-invariant direct methods
Least-squares matrix: full	Secondary atom site location: difference Fourier map
*R*[*F*^2^ > 2σ(*F*^2^)] = 0.066	Hydrogen site location: inferred from neighbouring sites
*wR*(*F*^2^) = 0.173	H-atom parameters constrained
*S* = 0.93	*w* = 1/[σ^2^(*F*_o_^2^) + (0.076*P*)^2^ + 0.6885*P*] where *P* = (*F*_o_^2^ + 2*F*_c_^2^)/3
4170 reflections	(Δ/σ)_max_ < 0.001
263 parameters	Δρ_max_ = 0.34 e Å^−^^3^
0 restraints	Δρ_min_ = −0.39 e Å^−^^3^

### Special details

**Table:**

Geometry. All s.u.'s (except the s.u. in the dihedral angle between two l.s. planes) are estimated using the full covariance matrix. The cell s.u.'s are taken into account individually in the estimation of s.u.'s in distances, angles and torsion angles; correlations between s.u.'s in cell parameters are only used when they are defined by crystal symmetry. An approximate (isotropic) treatment of cell s.u.'s is used for estimating s.u.'s involving l.s. planes.
Refinement. Refinement of F^2^ against ALL reflections. The weighted R-factor wR and goodness of fit S are based on F^2^, conventional R-factors R are based on F, with F set to zero for negative F^2^. The threshold expression of F^2^ > 2σ(F^2^) is used only for calculating R-factors(gt) etc. and is not relevant to the choice of reflections for refinement. R-factors based on F^2^ are statistically about twice as large as those based on F, and R- factors based on ALL data will be even larger.

### Fractional atomic coordinates and isotropic or equivalent isotropic displacement parameters (Å^2^)

**Table:**

	*x*	*y*	*z*	*U*_iso_*/*U*_eq_	Occ. (<1)
S1	0.30579 (11)	0.36333 (9)	0.10093 (6)	0.0571 (3)	
N1	0.3525 (3)	0.4674 (2)	−0.09649 (16)	0.0408 (5)	
N2	0.1316 (3)	0.6205 (3)	−0.01435 (19)	0.0522 (6)	
O1	0.6747 (4)	0.0390 (3)	0.0353 (3)	0.0991 (9)	
O2	0.0460 (5)	0.9217 (3)	−0.3230 (3)	0.1344 (14)	
O3	0.2781 (4)	0.7833 (3)	−0.37460 (18)	0.0887 (9)	
O4	0.2791 (3)	0.3934 (3)	−0.53399 (17)	0.0815 (7)	
C1	−0.0158 (5)	0.8666 (4)	−0.0993 (3)	0.0738 (10)	
H1A	0.0392	0.9470	−0.1201	0.111*	
H1B	−0.0530	0.8581	−0.0244	0.111*	
H1C	−0.1128	0.8870	−0.1457	0.111*	
C2	0.4657 (4)	0.2623 (3)	0.0315 (2)	0.0505 (7)	
C3	0.4737 (3)	0.3342 (3)	−0.0718 (2)	0.0448 (6)	
C4	0.5672 (5)	0.1135 (4)	0.0842 (3)	0.0678 (9)	
C5	0.3089 (3)	0.5520 (3)	−0.2070 (2)	0.0422 (6)	
H5	0.4132	0.5710	−0.2444	0.051*	
C6	0.1873 (4)	0.6996 (3)	−0.2021 (2)	0.0474 (7)	
C7	0.1055 (4)	0.7250 (3)	−0.1109 (2)	0.0505 (7)	
C8	0.5262 (6)	0.0589 (5)	0.1991 (3)	0.0958 (14)	
H8A	0.4158	0.0385	0.2040	0.144*	
H8B	0.5249	0.1339	0.2392	0.144*	
H8C	0.6109	−0.0315	0.2296	0.144*	
C9	0.2502 (3)	0.5029 (3)	−0.0135 (2)	0.0435 (6)	
C10	0.314 (4)	0.886 (2)	−0.4736 (17)	0.116 (7)	0.48 (3)
H10A	0.4280	0.8999	−0.4711	0.139*	0.48 (3)
H10B	0.2317	0.9832	−0.4829	0.139*	0.48 (3)
C10'	0.224 (2)	0.8853 (18)	−0.4809 (13)	0.085 (4)	0.52 (3)
H10C	0.1119	0.8797	−0.5001	0.102*	0.52 (3)
H10D	0.2164	0.9880	−0.4777	0.102*	0.52 (3)
C11	0.300 (2)	0.8136 (19)	−0.5624 (8)	0.102 (6)	0.48 (3)
H11A	0.4102	0.7506	−0.5754	0.152*	0.48 (3)
H11B	0.2622	0.8889	−0.6279	0.152*	0.48 (3)
H11C	0.2199	0.7537	−0.5422	0.152*	0.48 (3)
C11'	0.339 (2)	0.8426 (2)	−0.5548 (10)	0.182 (14)	0.52 (3)
H11D	0.4517	0.8313	−0.5285	0.273*	0.52 (3)
H11E	0.3191	0.9172	−0.6223	0.273*	0.52 (3)
H11F	0.3308	0.7485	−0.5669	0.273*	0.52 (3)
C12	0.2372 (2)	0.4638 (2)	−0.26787 (15)	0.0422 (6)	
C13	0.2885 (2)	0.4605 (3)	−0.37231 (16)	0.0495 (7)	
H13	0.3699	0.5105	−0.4044	0.059*	
C14	0.2219 (4)	0.3846 (3)	−0.4306 (2)	0.0561 (8)	
C15	0.1116 (4)	0.3055 (4)	−0.3822 (3)	0.0669 (9)	
H15	0.0690	0.2502	−0.4208	0.080*	
C16	0.0622 (4)	0.3063 (4)	−0.2764 (3)	0.0648 (9)	
H16	−0.0144	0.2515	−0.2432	0.078*	
C17	0.1232 (4)	0.3860 (3)	−0.2192 (2)	0.0504 (7)	
H17	0.0876	0.3875	−0.1474	0.060*	
C18	0.2065 (6)	0.3271 (5)	−0.6001 (3)	0.0995 (15)	
H18A	0.2330	0.2206	−0.5711	0.149*	
H18B	0.2517	0.3475	−0.6731	0.149*	
H18C	0.0847	0.3676	−0.6019	0.149*	
C19	0.1592 (5)	0.8125 (4)	−0.3027 (3)	0.0715 (10)	
C20	0.5962 (4)	0.2884 (4)	−0.1555 (3)	0.0626 (8)	
H20A	0.6810	0.1991	−0.1230	0.094*	
H20B	0.6506	0.3673	−0.1864	0.094*	
H20C	0.5375	0.2686	−0.2122	0.094*	

### Atomic displacement parameters (Å^2^)

**Table:**

	*U*^11^	*U*^22^	*U*^33^	*U*^12^	*U*^13^	*U*^23^
S1	0.0654 (5)	0.0657 (5)	0.0413 (4)	−0.0239 (4)	0.0025 (3)	−0.0068 (3)
N1	0.0435 (12)	0.0428 (11)	0.0375 (11)	−0.0094 (10)	0.0013 (9)	−0.0143 (9)
N2	0.0498 (14)	0.0546 (14)	0.0562 (15)	−0.0136 (12)	0.0120 (11)	−0.0231 (12)
O1	0.086 (2)	0.0692 (16)	0.123 (2)	0.0134 (15)	−0.0095 (18)	−0.0203 (16)
O2	0.163 (3)	0.090 (2)	0.097 (2)	0.038 (2)	−0.022 (2)	0.0100 (17)
O3	0.145 (3)	0.0573 (14)	0.0516 (14)	−0.0198 (15)	0.0099 (15)	0.0022 (11)
O4	0.1022 (19)	0.0988 (18)	0.0448 (13)	−0.0126 (15)	−0.0014 (12)	−0.0338 (12)
C1	0.065 (2)	0.0558 (19)	0.103 (3)	−0.0046 (17)	0.004 (2)	−0.0358 (19)
C2	0.0505 (17)	0.0482 (15)	0.0546 (17)	−0.0154 (14)	−0.0096 (13)	−0.0094 (13)
C3	0.0438 (15)	0.0420 (14)	0.0518 (16)	−0.0090 (12)	−0.0074 (12)	−0.0172 (12)
C4	0.063 (2)	0.060 (2)	0.082 (2)	−0.0211 (18)	−0.0222 (19)	−0.0070 (18)
C5	0.0461 (15)	0.0435 (14)	0.0367 (14)	−0.0118 (12)	0.0024 (11)	−0.0088 (11)
C6	0.0532 (17)	0.0395 (14)	0.0509 (16)	−0.0122 (13)	−0.0054 (13)	−0.0114 (12)
C7	0.0480 (17)	0.0445 (15)	0.0637 (19)	−0.0122 (13)	0.0008 (14)	−0.0215 (14)
C8	0.105 (3)	0.091 (3)	0.083 (3)	−0.042 (3)	−0.031 (2)	0.025 (2)
C9	0.0454 (15)	0.0511 (15)	0.0408 (14)	−0.0191 (13)	0.0057 (12)	−0.0176 (12)
C10	0.156 (18)	0.101 (8)	0.068 (9)	−0.029 (14)	0.018 (13)	0.017 (6)
C10'	0.103 (9)	0.066 (5)	0.064 (6)	−0.013 (7)	−0.008 (7)	0.020 (4)
C11	0.139 (12)	0.113 (10)	0.039 (7)	−0.022 (9)	−0.009 (7)	0.001 (6)
C11'	0.185 (19)	0.136 (14)	0.127 (17)	0.035 (13)	0.078 (15)	0.052 (13)
C12	0.0430 (15)	0.0424 (13)	0.0394 (14)	−0.0039 (12)	−0.0023 (11)	−0.0133 (11)
C13	0.0559 (18)	0.0520 (16)	0.0394 (15)	−0.0100 (14)	0.0015 (13)	−0.0126 (12)
C14	0.0628 (19)	0.0617 (18)	0.0406 (16)	−0.0011 (16)	−0.0048 (14)	−0.0211 (14)
C15	0.070 (2)	0.068 (2)	0.072 (2)	−0.0101 (18)	−0.0140 (18)	−0.0388 (18)
C16	0.062 (2)	0.069 (2)	0.075 (2)	−0.0282 (18)	0.0025 (17)	−0.0256 (17)
C17	0.0510 (17)	0.0561 (16)	0.0472 (16)	−0.0147 (14)	0.0023 (13)	−0.0176 (13)
C18	0.150 (4)	0.093 (3)	0.052 (2)	−0.004 (3)	−0.023 (2)	−0.035 (2)
C19	0.100 (3)	0.0505 (19)	0.060 (2)	−0.0115 (19)	−0.015 (2)	−0.0099 (16)
C20	0.0558 (19)	0.0620 (18)	0.067 (2)	0.0026 (15)	−0.0006 (16)	−0.0283 (16)

### Geometric parameters (Å, º)

**Table:**

S1—C9	1.736 (3)	C8—H8C	0.9800
S1—C2	1.753 (3)	C10—C11	1.50 (4)
N1—C9	1.368 (3)	C10—H10A	0.9900
N1—C3	1.391 (3)	C10—H10B	0.9900
N1—C5	1.475 (3)	C10'—C11'	1.358 (19)
N2—C9	1.290 (4)	C10'—H10C	0.9900
N2—C7	1.391 (4)	C10'—H10D	0.9900
O1—C4	1.228 (4)	C11—H11A	0.9800
O2—C19	1.201 (4)	C11—H11B	0.9800
O3—C19	1.325 (4)	C11—H11C	0.9800
O3—C10	1.486 (18)	C11'—H11D	0.9800
O3—C10'	1.499 (15)	C11'—H11E	0.9800
O4—C14	1.370 (4)	C11'—H11F	0.9800
O4—C18	1.420 (4)	C12—C13	1.3835
C1—C7	1.502 (4)	C12—C17	1.389 (3)
C1—H1A	0.9800	C13—C14	1.388 (4)
C1—H1B	0.9800	C13—H13	0.9500
C1—H1C	0.9800	C14—C15	1.372 (5)
C2—C3	1.350 (4)	C15—C16	1.392 (5)
C2—C4	1.487 (4)	C15—H15	0.9500
C3—C20	1.491 (4)	C16—C17	1.382 (4)
C4—C8	1.493 (5)	C16—H16	0.9500
C5—C12	1.526 (3)	C17—H17	0.9500
C5—C6	1.527 (4)	C18—H18A	0.9800
C5—H5	1.0000	C18—H18B	0.9800
C6—C7	1.353 (4)	C18—H18C	0.9800
C6—C19	1.468 (4)	C20—H20A	0.9800
C8—H8A	0.9800	C20—H20B	0.9800
C8—H8B	0.9800	C20—H20C	0.9800
			
C9—S1—C2	91.44 (13)	C11—C10—H10B	110.9
C9—N1—C3	115.6 (2)	H10A—C10—H10B	108.9
C9—N1—C5	119.6 (2)	C11'—C10'—O3	107.3 (13)
C3—N1—C5	123.6 (2)	C11'—C10'—H10C	110.3
C9—N2—C7	116.3 (2)	O3—C10'—H10C	110.3
C19—O3—C10	127.2 (10)	C11'—C10'—H10D	110.3
C19—O3—C10'	108.8 (8)	O3—C10'—H10D	110.3
C14—O4—C18	117.5 (3)	H10C—C10'—H10D	108.5
C7—C1—H1A	109.5	C10'—C11'—H11D	109.5
C7—C1—H1B	109.5	C10'—C11'—H11E	109.5
H1A—C1—H1B	109.5	H11D—C11'—H11E	109.5
C7—C1—H1C	109.5	C10'—C11'—H11F	109.5
H1A—C1—H1C	109.5	H11D—C11'—H11F	109.5
H1B—C1—H1C	109.5	H11E—C11'—H11F	109.5
C3—C2—C4	127.6 (3)	C13—C12—C17	119.90 (16)
C3—C2—S1	111.2 (2)	C13—C12—C5	119.31 (16)
C4—C2—S1	121.2 (2)	C17—C12—C5	120.8 (2)
C2—C3—N1	112.5 (2)	C12—C13—C14	120.55 (19)
C2—C3—C20	127.7 (3)	C12—C13—H13	119.7
N1—C3—C20	119.8 (2)	C14—C13—H13	119.7
O1—C4—C2	121.9 (3)	O4—C14—C15	125.4 (3)
O1—C4—C8	122.3 (4)	O4—C14—C13	114.9 (3)
C2—C4—C8	115.8 (3)	C15—C14—C13	119.7 (3)
N1—C5—C12	110.26 (19)	C14—C15—C16	119.9 (3)
N1—C5—C6	108.5 (2)	C14—C15—H15	120.1
C12—C5—C6	112.6 (2)	C16—C15—H15	120.1
N1—C5—H5	108.5	C17—C16—C15	120.8 (3)
C12—C5—H5	108.5	C17—C16—H16	119.6
C6—C5—H5	108.5	C15—C16—H16	119.6
C7—C6—C19	121.2 (3)	C16—C17—C12	119.2 (3)
C7—C6—C5	122.3 (2)	C16—C17—H17	120.4
C19—C6—C5	116.5 (3)	C12—C17—H17	120.4
C6—C7—N2	122.2 (3)	O4—C18—H18A	109.5
C6—C7—C1	125.6 (3)	O4—C18—H18B	109.5
N2—C7—C1	112.1 (3)	H18A—C18—H18B	109.5
C4—C8—H8A	109.5	O4—C18—H18C	109.5
C4—C8—H8B	109.5	H18A—C18—H18C	109.5
H8A—C8—H8B	109.5	H18B—C18—H18C	109.5
C4—C8—H8C	109.5	O2—C19—O3	120.5 (3)
H8A—C8—H8C	109.5	O2—C19—C6	127.3 (4)
H8B—C8—H8C	109.5	O3—C19—C6	112.2 (3)
N2—C9—N1	127.9 (3)	C3—C20—H20A	109.5
N2—C9—S1	122.8 (2)	C3—C20—H20B	109.5
N1—C9—S1	109.3 (2)	H20A—C20—H20B	109.5
O3—C10—C11	104.5 (18)	C3—C20—H20C	109.5
O3—C10—H10A	110.9	H20A—C20—H20C	109.5
C11—C10—H10A	110.9	H20B—C20—H20C	109.5
O3—C10—H10B	110.9		
			
C9—S1—C2—C3	0.6 (2)	C3—N1—C9—S1	−0.6 (3)
C9—S1—C2—C4	−176.3 (2)	C5—N1—C9—S1	167.21 (17)
C4—C2—C3—N1	175.6 (3)	C2—S1—C9—N2	−178.8 (2)
S1—C2—C3—N1	−1.0 (3)	C2—S1—C9—N1	−0.02 (19)
C4—C2—C3—C20	−6.0 (5)	C19—O3—C10—C11	−122.6 (13)
S1—C2—C3—C20	177.3 (2)	C10'—O3—C10—C11	−64 (3)
C9—N1—C3—C2	1.1 (3)	C19—O3—C10'—C11'	−173.7 (13)
C5—N1—C3—C2	−166.2 (2)	C10—O3—C10'—C11'	52 (3)
C9—N1—C3—C20	−177.4 (2)	N1—C5—C12—C13	−136.62 (19)
C5—N1—C3—C20	15.4 (4)	C6—C5—C12—C13	102.0 (2)
C3—C2—C4—O1	0.5 (5)	N1—C5—C12—C17	42.6 (3)
S1—C2—C4—O1	176.9 (3)	C6—C5—C12—C17	−78.8 (3)
C3—C2—C4—C8	−177.6 (3)	C17—C12—C13—C14	2.6 (3)
S1—C2—C4—C8	−1.2 (4)	C5—C12—C13—C14	−178.2 (2)
C9—N1—C5—C12	−103.6 (2)	C18—O4—C14—C15	6.5 (5)
C3—N1—C5—C12	63.1 (3)	C18—O4—C14—C13	−175.5 (3)
C9—N1—C5—C6	20.1 (3)	C12—C13—C14—O4	178.1 (2)
C3—N1—C5—C6	−173.1 (2)	C12—C13—C14—C15	−3.8 (4)
N1—C5—C6—C7	−15.3 (3)	O4—C14—C15—C16	−179.6 (3)
C12—C5—C6—C7	107.0 (3)	C13—C14—C15—C16	2.5 (5)
N1—C5—C6—C19	166.4 (2)	C14—C15—C16—C17	−0.1 (5)
C12—C5—C6—C19	−71.2 (3)	C15—C16—C17—C12	−1.1 (5)
C19—C6—C7—N2	−179.1 (3)	C13—C12—C17—C16	−0.2 (4)
C5—C6—C7—N2	2.7 (4)	C5—C12—C17—C16	−179.4 (3)
C19—C6—C7—C1	−1.8 (5)	C10—O3—C19—O2	14.9 (17)
C5—C6—C7—C1	−180.0 (3)	C10'—O3—C19—O2	−11.8 (9)
C9—N2—C7—C6	6.6 (4)	C10—O3—C19—C6	−164.6 (16)
C9—N2—C7—C1	−171.1 (2)	C10'—O3—C19—C6	168.8 (8)
C7—N2—C9—N1	−0.9 (4)	C7—C6—C19—O2	−15.5 (6)
C7—N2—C9—S1	177.68 (19)	C5—C6—C19—O2	162.8 (4)
C3—N1—C9—N2	178.1 (2)	C7—C6—C19—O3	163.9 (3)
C5—N1—C9—N2	−14.1 (4)	C5—C6—C19—O3	−17.8 (4)

### Hydrogen-bond geometry (Å, º)

**Table:**

*D*—H···*A*	*D*—H	H···*A*	*D*···*A*	*D*—H···*A*
C18—H18*B*···S1^i^	0.98	2.87	3.822 (3)	162

Symmetry code: (i) *x*, *y*, *z*−1.
