# Supplementary figures and images for: Crystal structure of 2-acetyl-5-(3-methoxyphenyl)-3,7-dimethyl-5H-1,3-thiazolo[3,2-a]pyrimidine-6-carboxylate
Source: Acta Crystallogr Sect E Struct Rep Online. 2014 Oct 29;70(Pt 11):o1204. doi: 10.1107/S1600536814023162 (PMC4257338; doi:10.1107/S1600536814023162)

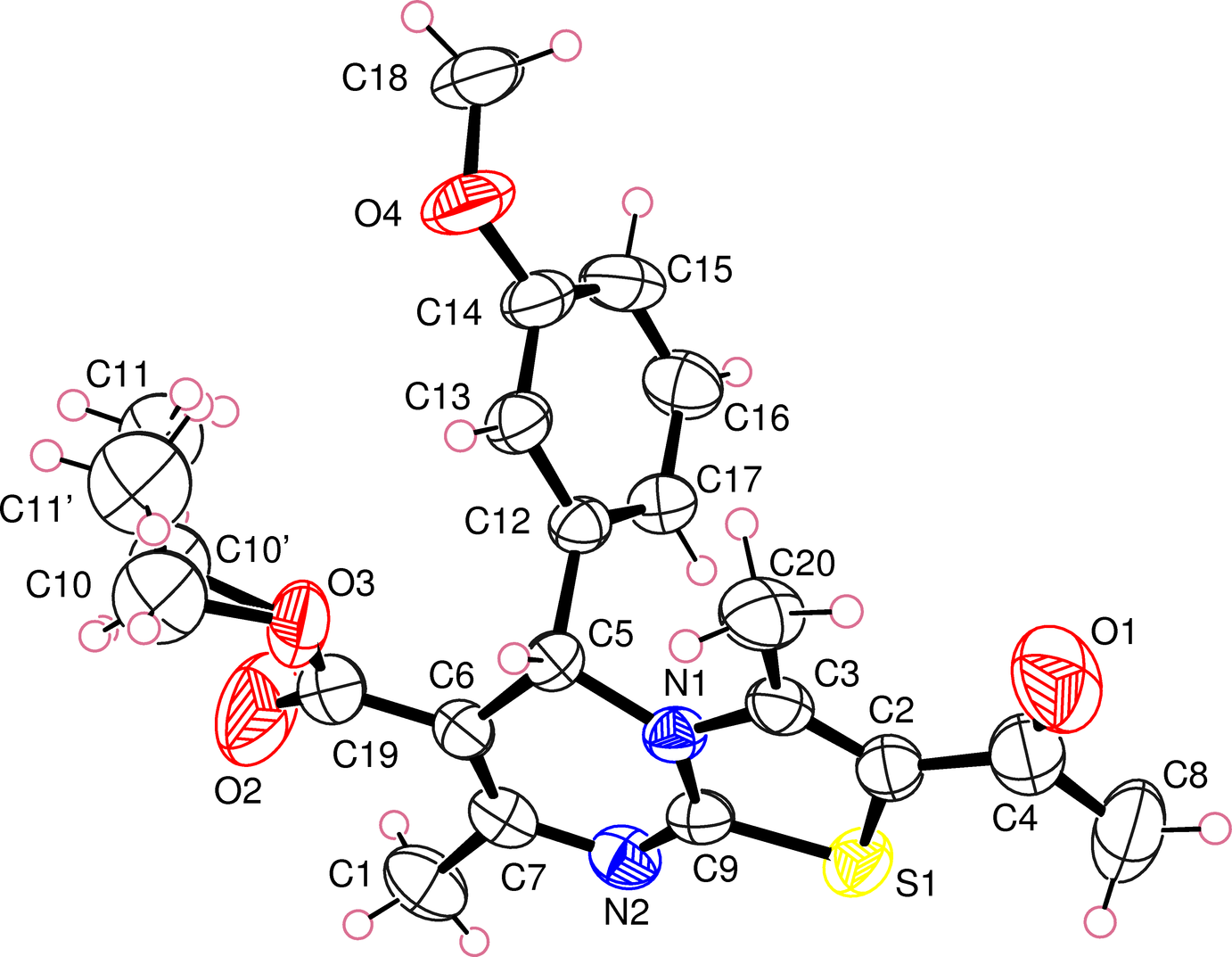

Supplement: Supplementary file 4 [file e-70-o1204-fig1.tif]

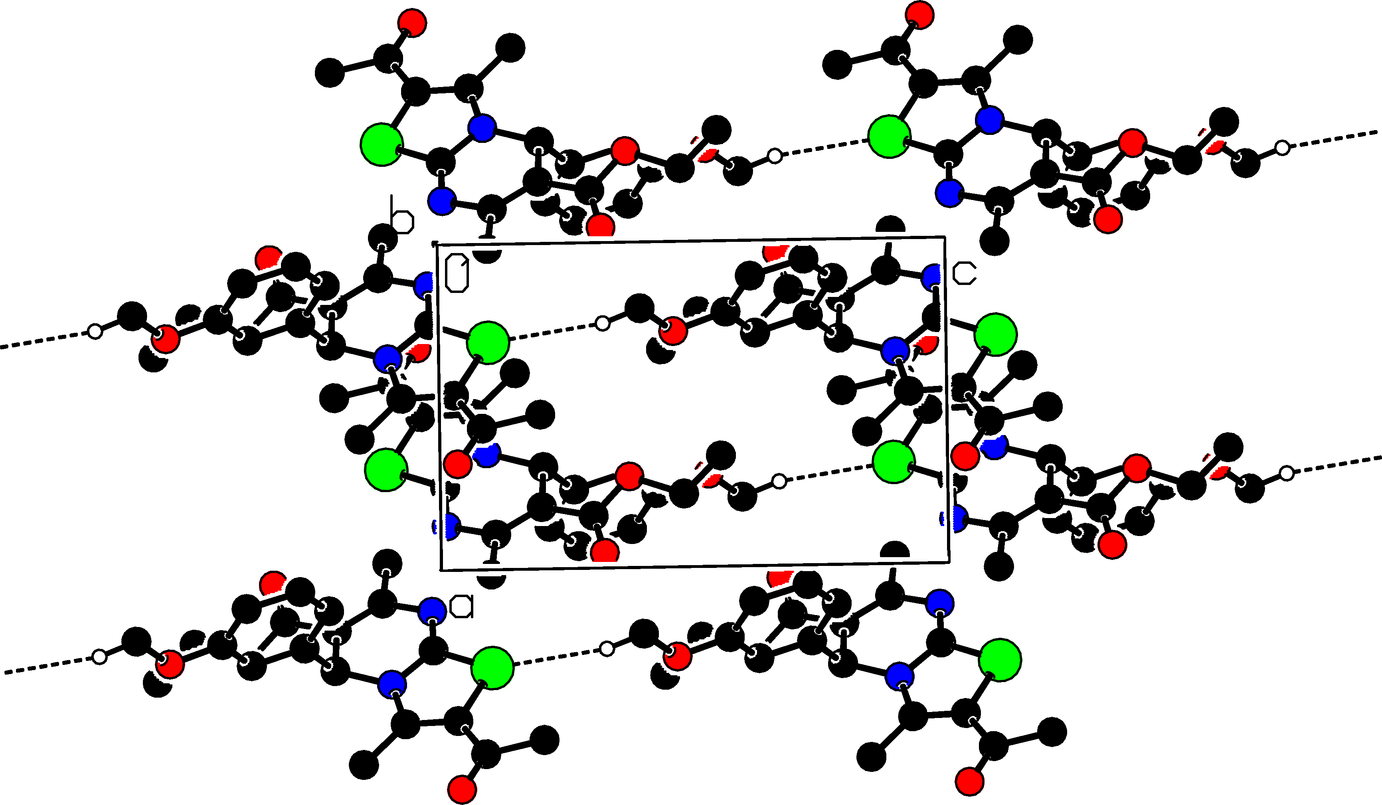

Supplement: Supplementary file 5 [file e-70-o1204-fig2.tif]
